# Macrodystrophia Lipomatosa: A Rare Cause of Bilateral Lower Limb Gigantism

**DOI:** 10.7759/cureus.18986

**Published:** 2021-10-23

**Authors:** Dijendra Nath Biswas, Arkadeep Dhali, Sabnam Parvin, Archana Singh, Gopal Krishna Dhali

**Affiliations:** 1 Department of Radiology, Institute of Post Graduate Medical Education and Research, Kolkata, IND; 2 Department of Gastrointestinal Surgery, Institute of Post Graduate Medical Education and Research, Kolkata, IND; 3 Department of Gastroenterology, Institute of Post Graduate Medical Education and Research, Kolkata, IND

**Keywords:** macrodystrophia lipomatosa, cosmetic surgery, fibrolipohamartoma, overgrowth syndrome, localised gigantism

## Abstract

Macrodystrophia lipomatosa (MDL) is a rare congenital overgrowth syndrome characterised by inadvertent proliferation of all the mesenchymal elements resulting in localised gigantism. Herein, we present an eight-month-old female child, who presented to us with a history of gradual enlargement of both lower limbs along with the toes which was noticed by the parents a few days after birth. There was no history of trauma, pain or skin changes. Physical examination revealed unusual hypertrophy of both feet and toes. It was non-tender with no evidence of oedema or bruit over the swelling. X-ray of lower limbs revealed bony hypertrophy and overgrowth of all the bones with increased soft tissue shadow of bilateral foot. On ultrasound evaluation of the lower limbs, there was increased soft tissue in both dorsal and plantar aspect of bilateral foot without any vascular malformation. To characterise the swelling better, magnetic resonance imaging was warranted which revealed accumulation of excessive fat in the subcutaneous tissue without discernible capsule. Fibrous strand within the fat in bilateral feet, both in the plantar and dorsal aspect (more in plantar aspect), was seen. Core tissue biopsy was performed which showed abundant adipose tissue dispersed in mesh-like fibrous tissue and infiltrating the dermal connecting, suggestive of macrodystrophia lipomatosa. Currently, patient is advised for corrective surgery. Clinicians should be aware of these atypical presentations of MDL to differentiate it from other causes of local gigantism like fibrolipohamartoma (FLH) of nerve sheath, lymphangiomatosis, hemangiomatosis, Proteus syndrome, Klippel-Trenaunay syndrome and neurofibromatosis 1 as they differ in management and outcome.

## Introduction

Macrodystrophia lipomatosa (MDL) is a rare congenital disorder of local gigantism by the overgrowth of mesenchymal elements (including the periosteum, bone marrow, nerve sheath or muscle), predominantly the fibroadipose tissue [1,2]. The affected limb increases in length and girth until the child reaches puberty. Usually, involvement of unilateral foot with predilection for second and third digits is commonly encountered [3,4]. In our case, bilateral involvement of lower limbs along with involvement of all the digits was seen. It is important to report such anecdotal cases to get knowledge about their clinicopathological behaviour and standardise optimal treatment options since prospective studies are not feasible due to rarity of the disease and paucity of data.

## Case presentation

**Figure 1:**
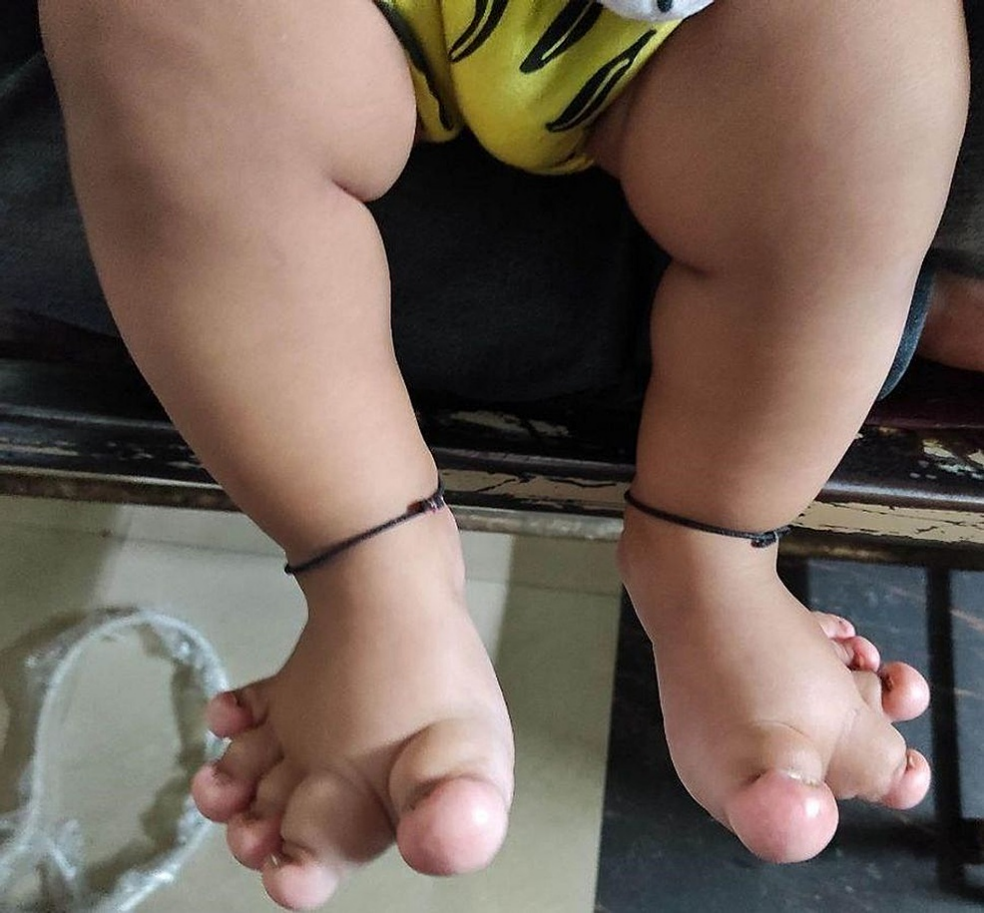
Abnormal enlargement of bilateral feet and all the toes.

Herein, we present an eight-month-old female child born out of non-consanguineous marriage via normal vaginal delivery, who presented to us with a history of gradual enlargement of both lower limbs along with the toes which was noticed by the parents a few days after birth. This was not associated with any delayed attainment of developmental milestones. There was no history of trauma, pain or skin changes. There is no relevant family history of any similar diseases. The upper limbs were normal. There were no stigmata of neurofibromatosis. On further reviewing the reports of antenatal ultrasound, no limb asymmetry was documented. Physical examination revealed unusual hypertrophy of both feet and toes (Figure 1).

**Figure 2:**
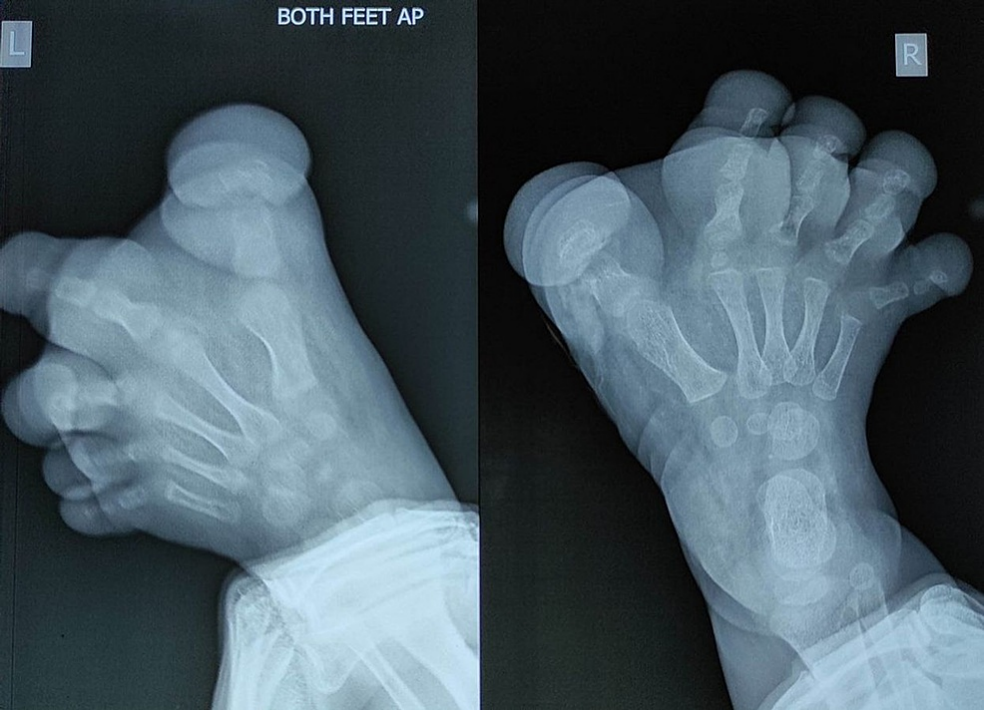
X-ray AP view of bilateral feet showing bony hypertrophy and overgrowth of all the bones with increased soft tissue shadow. AP: anteroposterior, L: left, R: right.

**Figure 3:**
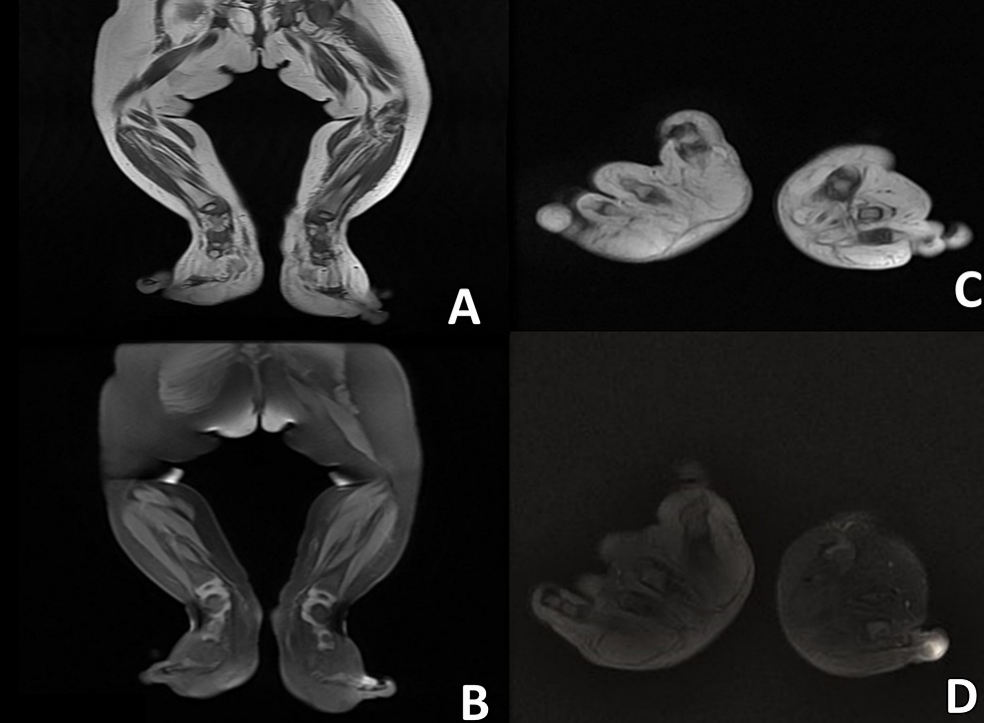
MRI image. Coronal T1W (A), coronal fat-suppressed sequence (B), axial T1W (C) and axial T1 fat suppressed (D) showing accumulation of excessive fat in the subcutaneous tissue without discernible capsule. Fibrous strands are seen within the fat in bilateral feet both in plantar and dorsal aspect (more in plantar aspect). There is osseous hypertrophy and bony overgrowth without any cortical thickening

The skin over this was thickened. It was non-tender with no evidence of oedema or bruit over the swelling. X-ray of lower limbs revealed bony hypertrophy and overgrowth of all the bones with increased soft tissue shadow of bilateral foot (Figure 2). No evidence of osteolytic or sclerotic lesions was seen, and no periosteal reaction was noted. On ultrasound evaluation of the lower limbs, there was increased soft tissue in both dorsal and plantar aspect of bilateral foot without any vascular malformation. To characterise the swelling better, magnetic resonance imaging (Figure 3A-3D) was warranted which revealed accumulation of excessive fat in the subcutaneous tissue without discernible capsule. Fibrous strand within the fat in bilateral feet, both in the plantar and dorsal aspect (more in plantar aspect), was seen. There was osseous hypertrophy and bony overgrowth without any cortical thickening. Core tissue biopsy was performed which showed abundant adipose tissue dispersed in mesh-like fibrous tissue and infiltrating the dermal connecting, suggestive of macrodystrophia lipomatosa. Currently, patient is advised for corrective surgery. 

## Discussion

Macrodystrophia lipomatosa (MDL) is a rare congenital overgrowth syndrome characterised by inadvertent proliferation of all the mesenchymal elements that result in localised gigantism in the form of overgrowth of affected limbs [1,2]. It has no variation in sex distribution, and the exact incidence is unknown. Inglis has hypothesised probable theories for pathogenesis which include (1) humoral mechanism, (2) vascular mechanism and (3) neural mechanism [5]. PIK3CA gene association has been highlighted in newer studies [6]. Unilateral foot involvement with predilection for second and third digits is commonly encountered [3,4]. In our case, all the digits of both feet were involved. This was unique to our case as generalised involvement of all the digits of both lower limbs in MDL has never been reported before. 

Most of the cases have shown to increase in length as well as girth until puberty [7]. According to the pattern of growth, Barsky had classified MDL into static and progressive type [8]. In terms of diagnostic imaging modality, plain radiograph, magnetic resonance imaging (MRI) and ultrasound evaluation are used [9]. MRI can demonstrate diffuse fatty overgrowth without discernible capsule in the subcutaneous area. It is also helpful in picking up abnormalities of the neurovascular components [10,11]. Definitive diagnosis of MDL is quite challenging due to its similarities with other disorders like neurofibromatosis 1 (NF-1), fibrolipohamartoma (FLH) of nerve sheath, lymphangiomatosis, hemangiomatosis, Proteus syndrome and Klippel-Trenaunay syndrome. FLH presents with isolated nerve involvement associated with intramuscular fat deposition, contrary to MDL, where all components of the mesenchymal elements are involved [12]. NF-1 is usually bilateral with other associated findings like café-au-lait spot and multiple cutaneous neurofibromas [13]. Proteus syndrome presents with skull abnormalities, pigmented nevi, lung cysts and intraabdominal lipoma [14]. Port-wine stain and varicose veins are seen in Klippel-Trenaunay syndrome [15]. 

Surgical intervention is required in MDL. The principle over here is to improve cosmetic appearance with utmost care in preserving the physiological functions. Multiple debulking surgeries with or without partial amputation have shown to provide promising results. In absence of neurological signs and symptoms, surgery should be delayed till completion of patient’s growth [16].

## Conclusions

What is unique about our case was the bilateral involvement of lower limbs along with involvement of all the digits. It is important to report such anecdotal cases to get knowledge about their clinicopathological behaviour and standardise optimal treatment options since prospective studies are not feasible due to rarity of the disease and paucity of data. Moreover, clinicians should be aware of these atypical presentations to differentiate it from other causes of local gigantism like fibrolipohamartoma (FLH) of nerve sheath, lymphangiomatosis, hemangiomatosis, Proteus syndrome, Klippel-Trenaunay syndrome and neurofibromatosis 1 as they differ in management and outcome.
